# ‘Incidental thyroid cancer’ is not synonymous with ‘overdiagnosis’

**DOI:** 10.1530/ETJ-24-0283

**Published:** 2024-10-14

**Authors:** Oleksiy Tsybrovskyy, Manuel Sobrinho-Simões, Giovanni Tallini

**Affiliations:** 1 Diagnostic and Research Institute of Pathology, Medical University of Graz, Austria; 2 Department of Molecular Pathology and Immunology of the University of Porto, Portugal; 3 Head of the Endocrine Pathology program, University of Bologna Medical Center, Bologna, Italy

Dear Editor,

We read with great interest the paper by Cosme and co-workers about the differences between incidentally (ITC) and non-incidentally (NITC) diagnosed papillary thyroid carcinoma (PTC) (1). We congratulate the authors for the excellent work-up of the study cohort that comprised 122 ITCs and 103 NITCs. Intriguingly, the study revealed no significant difference between ITC and NITC groups regarding the pTNM staging, American Thyroid Association (ATA) recurrence risk, proportion of aggressive PTC subtypes, frequency of radioactive iodine (RAI) prescription, and RAI activity. Moreover, the diagnostic modality (incidental vs symptomatic) had no impact on disease persistence at the 5-year follow-up both on univariate and on multivariate analyses. Although ITCs were smaller and of lower pT status than NITCs (which was expected given the definition of the groups), a considerable proportion of ITCs (36%) were yet larger than 2 cm in size.

Based on these results, the authors are trying to address in their paper the ongoing debate as to whether the worldwide rise in TC incidence is mainly attributed to overdiagnosis (2) or to some objective factors like environmental exposures and lifestyle (3, 4). In particular, they conclude that ‘as even large tumours can be ITC, overdiagnosis is the most likely cause of increasing incidence of TC’. The authors seem to equate carcinomas found incidentally (ITC) with ‘overdiagnosis’. However, we believe this is not appropriate and actually not supported by the results of the study.

Overdiagnosis refers to a condition that, if unrecognized, would not cause symptoms or harm a patient during his or her life (5). In other words, an ‘overdiagnosed’ condition (including cancer) must be bothincidentaland harmless. True incidentalomas (i.e. TCs diagnosed incidentally at the histological examination of benign thyroid conditions) are the perfect example, as they almost never recur (6). This case of ‘overdiagnosis’ is, however, well recognized and does not lead to overtreatment, since most incidentalomas do not require any additional therapy (7). The ITCs in the study by Cosme and co-workers were, by contrast, not harmless, as only 61.5% of ITC patients were completely disease free at the 5-year follow-up and the course of the disease was not different from NITC patients. This finding is especially remarkable given the fact that true histologic incidentalomas were also included in the ITC group. Strictly speaking, the results of the study indicate that increased TC detection by clinical imaging is notoverdetection (which is the most frequent source of overdiagnosis (8)). Indeed, the study suggests that increased use of imaging may lead to the identification of clinically relevant tumors that could potentially harm the patient. Since the authors have included in the cohort ‘TC diagnosed incidentally at the histological examination of benign thyroid lesions’, they may want to analyze the clinicopathologic features of these tumors vs the incidental tumors found by imaging and the non-incidental ones.

The matter of TC overdiagnosis, however, is more complicated, as it includes, besides overdetection, overdefinition (8). The most prominent example of TC cancer overdefinition is certainly the ‘Non-invasive encapsulated follicular variant of PTC’. This entity has been routinely diagnosed by pathologists over decades as carcinoma in spite of its indolent nature. Only in 2017 – in the 4th edition of the WHO classification of endocrine tumors – it was downgraded to NIFTP, an essentially benign condition (9). This evidence-based terminology change alone may have reduced TC diagnoses by up to 20%, particularly in the United States (10, 11). It is not clear to what extent the study by Cosme and co-workers is affected by TC overdefinition because no histopathological review and re-classification of the cases were undertaken. In any case, the overdefinition of the non-invasive encapsulated follicular variant of PTC would have equally affected both ITC and NITC.

## Declaration of interest

The authors declare that there is no conflict of interest that could be perceived as prejudicing the impartiality of the study reported.

## Funding

This work did not receive any specific grant from any funding agency in the public, commercial, or not-for-profit sector.
